# TTFs nonsymmetrically fused with alkylthiophenic moieties

**DOI:** 10.3762/bjoc.11.71

**Published:** 2015-05-05

**Authors:** Rafaela A L Silva, Bruno J C Vieira, Marta M Andrade, Isabel C Santos, Sandra Rabaça, Dulce Belo, Manuel Almeida

**Affiliations:** 1 Centro de Ciências e Tecnologias Nucleares, Campus Tecnológico e Nuclear, Instituto Superior Técnico, Universidade de Lisboa, Estrada Nacional 10, ao km 139,7, 2695-066 Bobadela LRS, Portugal

**Keywords:** cyclic voltammetry, single-crystal X-ray diffraction, supramolecular chemistry, tetrathiafulvalene (TTF), thiophene

## Abstract

Two new dithiolene ligand precursors, containing fused TTF and alkyl thiophenic moieties 3,3'-{[2-(5-(*tert*-butyl)thieno[2,3-*d*][1,3]dithiol-2-ylidene)-1,3-dithiole-4,5-diyl]bis[sulfanediyl]}dipropanenitrile (α-tbtdt, **1**), and 3,3'-{[2-(5-methylthieno[2,3-*d*][1,3]dithiol-2-ylidene)-1,3-dithiole-4,5-diyl]bis[sulfanediyl]}dipropanenitrile (α-mtdt, **2**), were synthesized and characterized. The electrochemical properties of these electronic donors were studied by cyclic voltammetry (CV) in dichloromethane. Both compounds show two quasi-reversible oxidation processes, versus Ag/AgCl, typical of TTF donors at *E*^1^_1/2_ = 279 V and *E*^2^_1/2_ = 680 V for **1** and *E*^1^_1/2_ = 304 V and *E*^2^_1/2_ = 716 V in the case of **2**. The single-crystal X-ray structure of **1** and of a charge transfer salt of **2**, (α-mtdt)[Au(mnt)_2_] (**3**), are reported.

## Introduction

Since the discovery of the first organic metals and superconductors the field of electronic molecular materials has been largely dominated by derivatives of the organic donor tetrathiafulvalene (TTF) [1]. More than one thousand TTF derivatives have been reported in the last 40 years and many have been at the basis of several conducting, superconducting and other important electronic materials [2–6]. Bisdithiolene–transition metal complexes with square planar structures can be seen as inorganic TTF analogues, in which a transition metal replaces the central double bond. They have similar frontier orbitals to TTF and have been also at the basis of several electronic materials [7]. An additional connection between the TTF derivatives and the bisdithiolene–transition metal complexes was recently provided by complexes with dithiolene ligands incorporating TTF units, which have been at the basis of several highly conducting materials based on a single neutral molecular species [8].

Among the highly extended ligands with TTF moieties, those that also contain thiophenic units have led to a family of complexes with interesting transport and magnetic properties [9–10]. A remarkable example is the neutral complex [Ni(dtdt)_2_] (dtdt = 3-{5-[(2-cyanoethyl)thio]-2-(5,6-dihydrothieno[2,3-*d*][1,3]dithiol-2-ylidene-1,3-dithiol-4-yl)thio}propanenitrile), which presents transport properties typical of a metallic system, even when measured in a polycrystalline sample, with an electrical conductivity at room temperature of 200 S/cm. These complexes have been prepared from cyanoethyl-substituted TTF-thiophenedithiolates [9,11]. One of the limitations in the study of these complexes and their possible applications is their low solubility mainly in the neutral state. This limitation can be, in principle, overcome by appropriated functionalization of the ligands as already explored for less extended complexes [12].

**Scheme 1:**
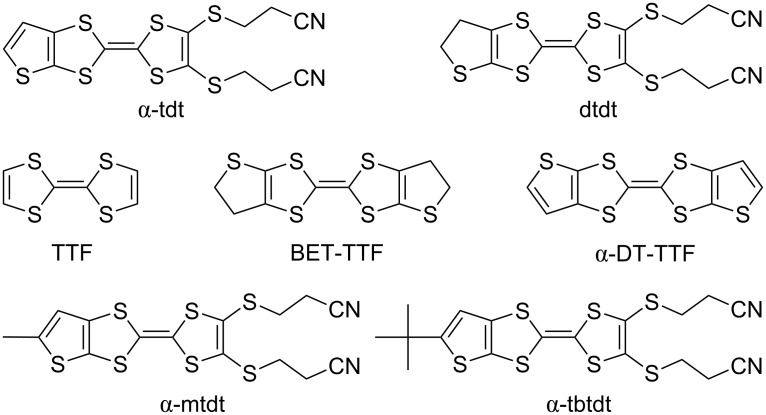
Molecular diagrams of α-tbtdt (**1**) and α-mtdt (**2**), and related TTF-type donors.

Here we report synthesis and characterization of two new sulfur-rich TTF type donors annulated to alkylthiophene rings, 3,3'-{[2-(5-(*tert*-butyl)thieno[2,3-*d*][1,3]dithiol-2-ylidene)-1,3-dithiole-4,5-diyl]bis[sulfanediyl]}dipropanenitrile (α-tbtdt, **1**), and 3,3'-{[2-(5-methylthieno[2,3-*d*][1,3]dithiol-2-ylidene)-1,3-dithiole-4,5-diyl]bis[sulfanediyl]}dipropanenitrile (α-mtdt, **2**) (Scheme 1). These new TTF-type donors can also be converted to transition metal complexes based on extended thiophene/TTF-fused dithiolene ligands. They were obtained by cross-coupling reactions between alkylated thio and oxo compounds. The incorporation of these alkyl groups in the thiophenic ring is expected to increase the solubility of these TTFs and analogous transition metals complexes, enabling their processing from solutions.

## Results and Discussion

### Synthesis

**Scheme 2:**
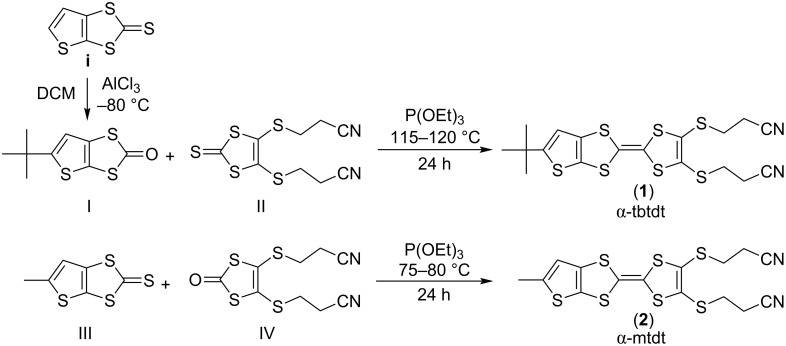
Synthetic route for compounds **1** and **2**.

The TTF donors fused non-symetrically with substituted thiophene moieties **1** and **2** were obtained by the cross coupling, in the presence of trimethyl phosphite, between compounds **I** and **II** and between compounds **III** and **IV**, respectively, following a general procedure for non-symmetrically substituted TTFs [13] as first described by Underhill and co-workers [14] (Scheme 2). 5-(*tert*-Butyl)thieno[2,3-*d*][1,3]dithiol-2-one (**I**) was prepared by a low temperature Friedel–Crafts alkylation of 5,6-thieno[2,3-*d*]-1,3-dithiol-2-one (**i**), a new procedure with a better global yield than the one reported previously [15]. 4,5-Bis(2-cyanoethylthio)-1,3-dithiole-2-thione (**II**), was obtained as described before [16] from a bis(tetraethylammonium)bis(2-thioxo-1,3-dithiole-4,5-dithiolate)zincate complex [17], by nucleophilic substitution [14]. Compound **II** was converted in 4,5-bis(2-cyanoethylthio)-1,3-dithiol-2-one (**IV**), using mercuric acetate and acetic acid in chloroform. 5-methylthieno[2,3-*d*][1,3]dithiole-2-thione (**III**) was obtained through a multi-step reaction as previously reported [16].

The preparations of compounds **1** and **2** through the coupling reactions depicted in Scheme 2 also resulted in several byproducts, mainly from homo-coupling reactions. Reaction time, temperature, and amount of solvent were crucial factors for the final yields and for product purity. By optimizing these reaction parameters an acceptable yield of 28% for **1** and a very good yield of 63% for **2** could be obtained after purification through column chromatography, eluting with dichloromethane. The new TTF-type donors are soluble in commonly used organic solvents, such as CH_2_Cl_2_, CHCl_3_, acetonitrile and *n*-hexane.

Single crystals suitable for X-ray measurements, could be isolated by slow evaporation of chloroform and *n*-hexane solutions, for **1** and **I**, respectively. Compound **2** crystallizes, from *n*-hexane/CH_2_Cl_2_ (3:1), in very thin fibres too small for X-ray diffraction. However, thanks to its reasonable solubility in dichloromethane, a charge transfer salt of **2**, (α-mtdt)[Au(mnt)_2_] (**3**), (mnt = maleonitriledithiolate) could be obtained by electrocrystallization using standard conditions.

### Redox properties

**Figure 1:**
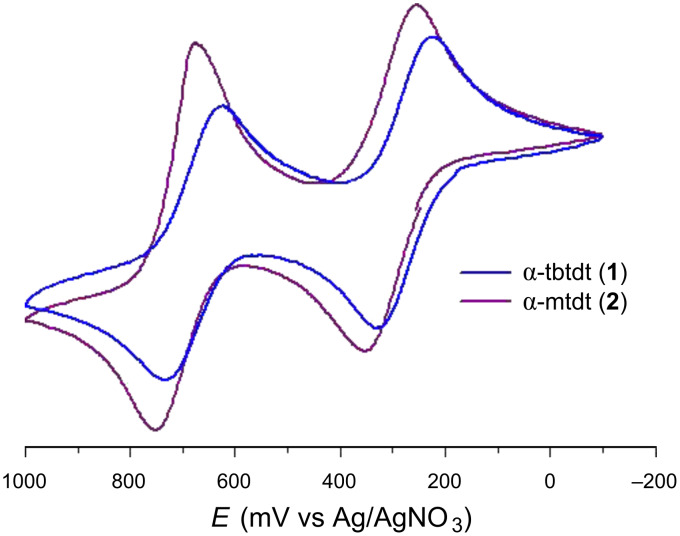
Cyclic voltammogram of α-tbtdt (**1**) and α-mtdt (**2**) (10^−3^ M) in dichloromethane versus Ag/AgNO_3_, with *n*-Bu_4_NPF_6_ (10^−1^ M) as supporting electrolyte. Scan rate *ν* = 100 mV/s at room temperature.

The redox properties of the donors **1** and **2** in solution were studied by cyclic voltammetry and the results are collected in Table 1 along with the closely related compounds (DT-TTF = dithiophene-tetrathiafulvalene, BET-TTF = bis(ethylenethio)-tetrathiafulvalene, α-DT-TTF = alpha-dithiophene-tetrathiafulvalene, dtdt = 3-{5-[(2-cyanoethyl)thio]-2-(5,6-dihydrothieno[2,3-*d*][1,3]dithiol-2-ylidene-1,3-dithiol-4-yl)thio}propanenitrile, α-tdt = 3-({5-[(2-cyanoethyl)thio]-2-thieno[2,3-*d*][1,3]dithiol-2-ylidene-1,3-dithiol-4-yl)thio}propanenitrile). Compounds **I** and **II** undergo two separate quasi-reversible one-electron oxidation processes typical of TTF-based donors (Figure 1). Cyclic voltammetry of α-tbtdt in dichloromethane shows a pair of processes at 0.680 V and 0.279 V, vs Ag/AgNO_3_, which are ascribed to the couples [α-tbtdt]^+^/[α-tbtdt]^2+^ and [α-tbtdt]^0^/[α-tbtdt]^+^, respectively. For α-mtdt, similar quasi-reversible waves are observed at 0.304 V and 0.716 V ascribed to the [α-mtdt]^0^/[α-mtdt]^+^ and [α-mtdt]^+^/[α-mtdt]^2+^ couples, respectively. These electrochemical studies show that α-tbtdt and α-mtdt are easier to oxidise than the related unsubstituted extended TTFs, with higher electron donor ability compared to related TTF-type donors with the exception of BET-TTF.

**Table 1:** Electrochemical data (oxidation potentials *E* and half wave potentials *E*_1/2_ for quasi reversible processes), vs Ag/AgNO_3_ in DCM, of TTF derivatives **1** and **2** as well as of the related donors.

donor	*E*_1, D_^0^_/D_^+^ (mV)	*E*_2, D_^+^_/D_^2+^ (mV)

BET-TTF [18]	215	650
α-DT-TTF [18]	320	730
DT-TTF [18]	542	1015

donor	*E*^1^_1/2, D_^0^_/D_^+^ (mV)	*E*^2^_1/2, D_^+^_/D_^2+^ (mV)

TTF [19]	370^a^	750^a^
dtdt [11]	639^a^	997^a^
α-tdt [11]	612^a^	906^a^
α-tbtdt (**1**)	279	680
α-mtdt (**2**)	304	716

^a^Data collected in acetonitrile.

### Crystal structures

#### Ketone I

**Figure 2:**
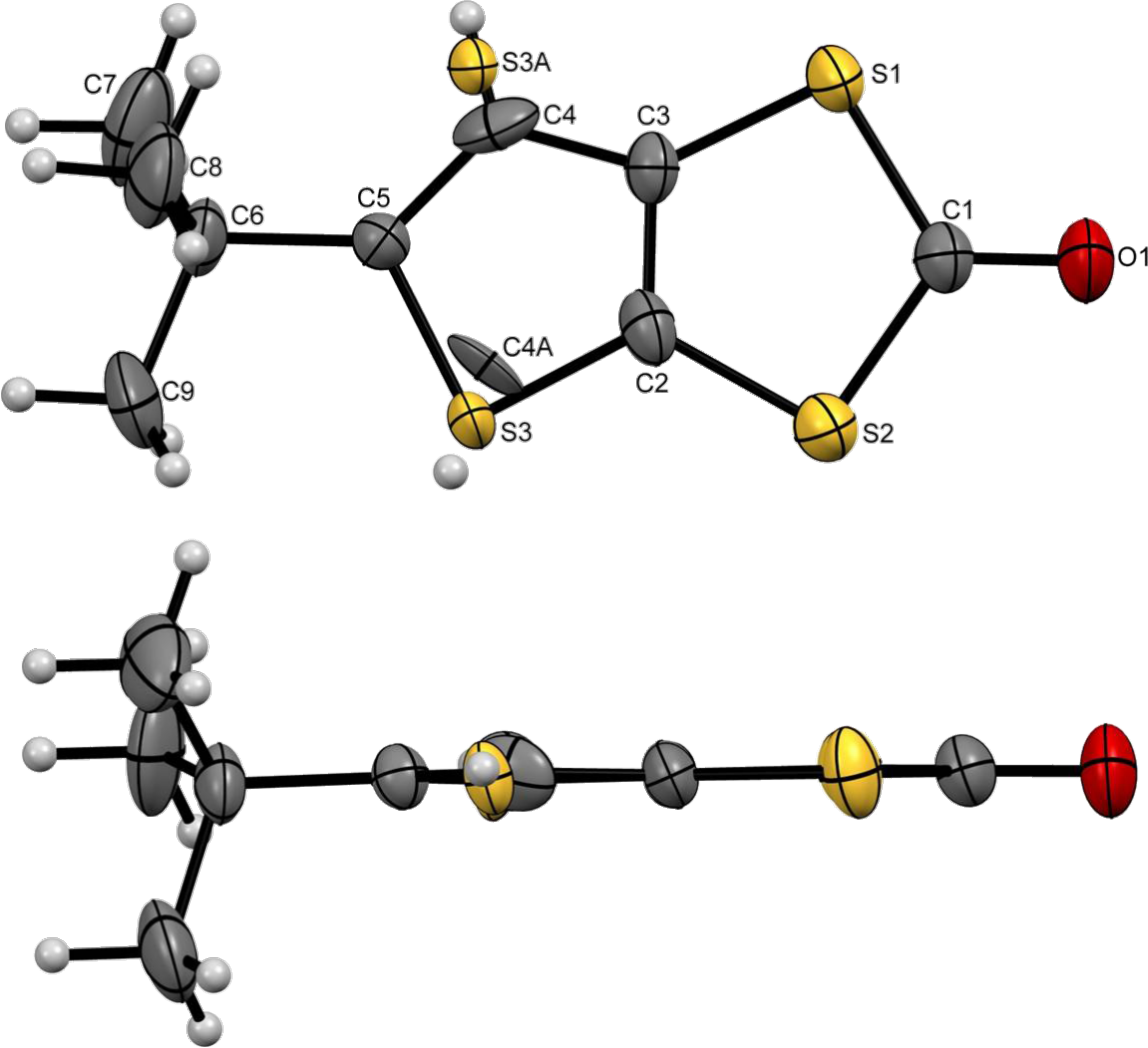
ORTEP view and atomic numbering scheme of **I** with thermal ellipsoids at 50% probability level.

Compound **I** crystallises in the orthorhombic system, space group *P*2_1_2_1_2_1_. The unit cell contains one independent molecule. The terminal sulfur atom presents an orientation disorder as denoted by two nearly identical occupation factors of 49% and 51% for S3 and S3A, respectively (Figure 2). This is most likely the result of orientation disorder of the molecule in the most stable trans configuration, rather than a cis–trans disorder. The bond lengths are within the expected range for thiophenic dithiol ketones [12,20].

**Figure 3:**
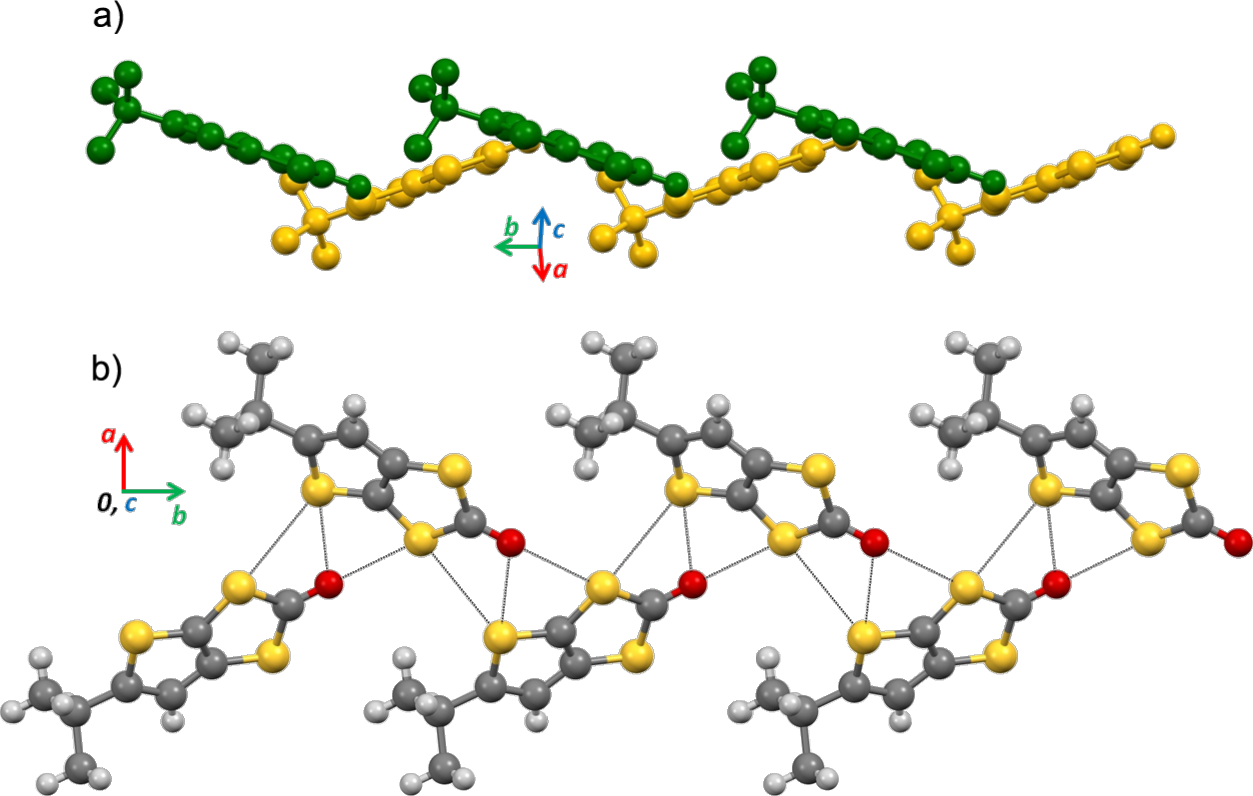
Molecular herringbone bi-chains in the crystal structure of **I**: a) view along the molecular planes and perpendicularly to the chain axis *b*, with molecules in different chains colored differently and b) view along *c*. The short contacts are represented as black dotted lines.

**Figure 4:**
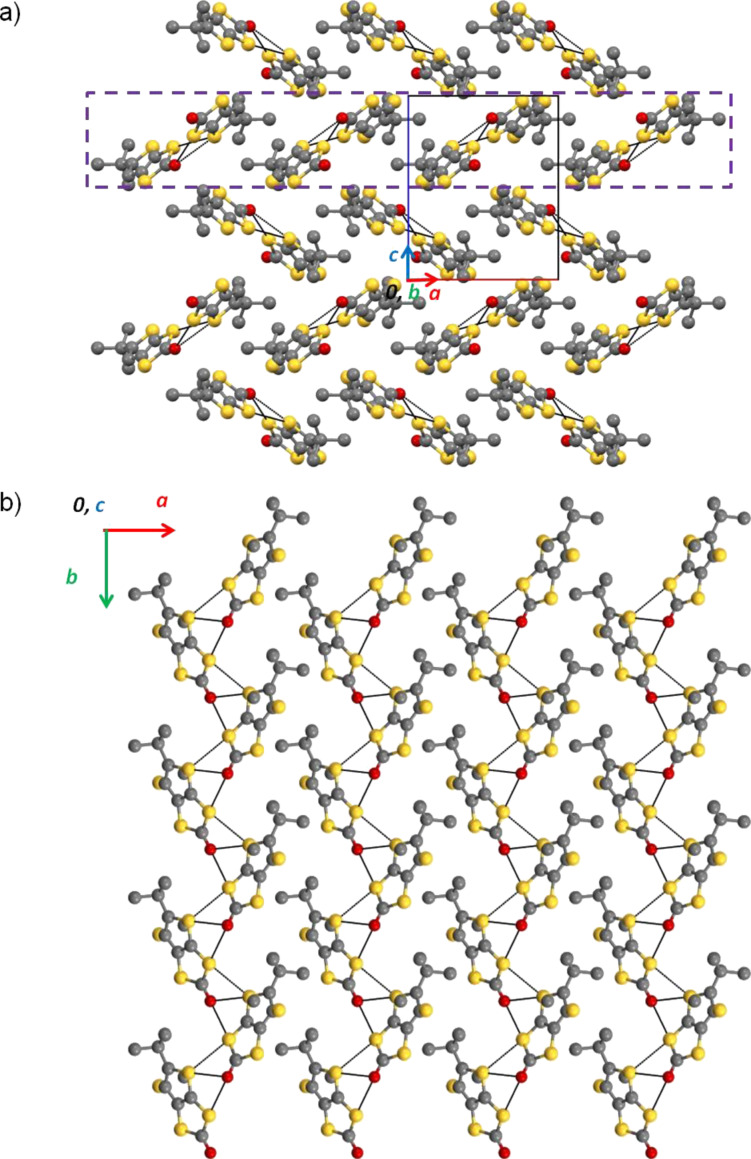
Crystal structure of **I** a) view along the *b* axis*,* b) Partial view of one layer of bi-chains in the *a,b* plane corresponding to the dashed line in a).

The crystal structure is composed of pairs of side-by-side chains of ketone **1** running parallel to *b*. Within these bi-chains, the molecules are connected by short S^…^S and S^…^O interactions with the *tert*-butyl groups pointing outside, in a herringbone fashion (Figure 3b). Molecules in different chains make an angle of circa 39° (Figure 3a). There are no close contacts between bi-chains (Figure 4).

#### α-tbtdt (**1**)

**Figure 5:**
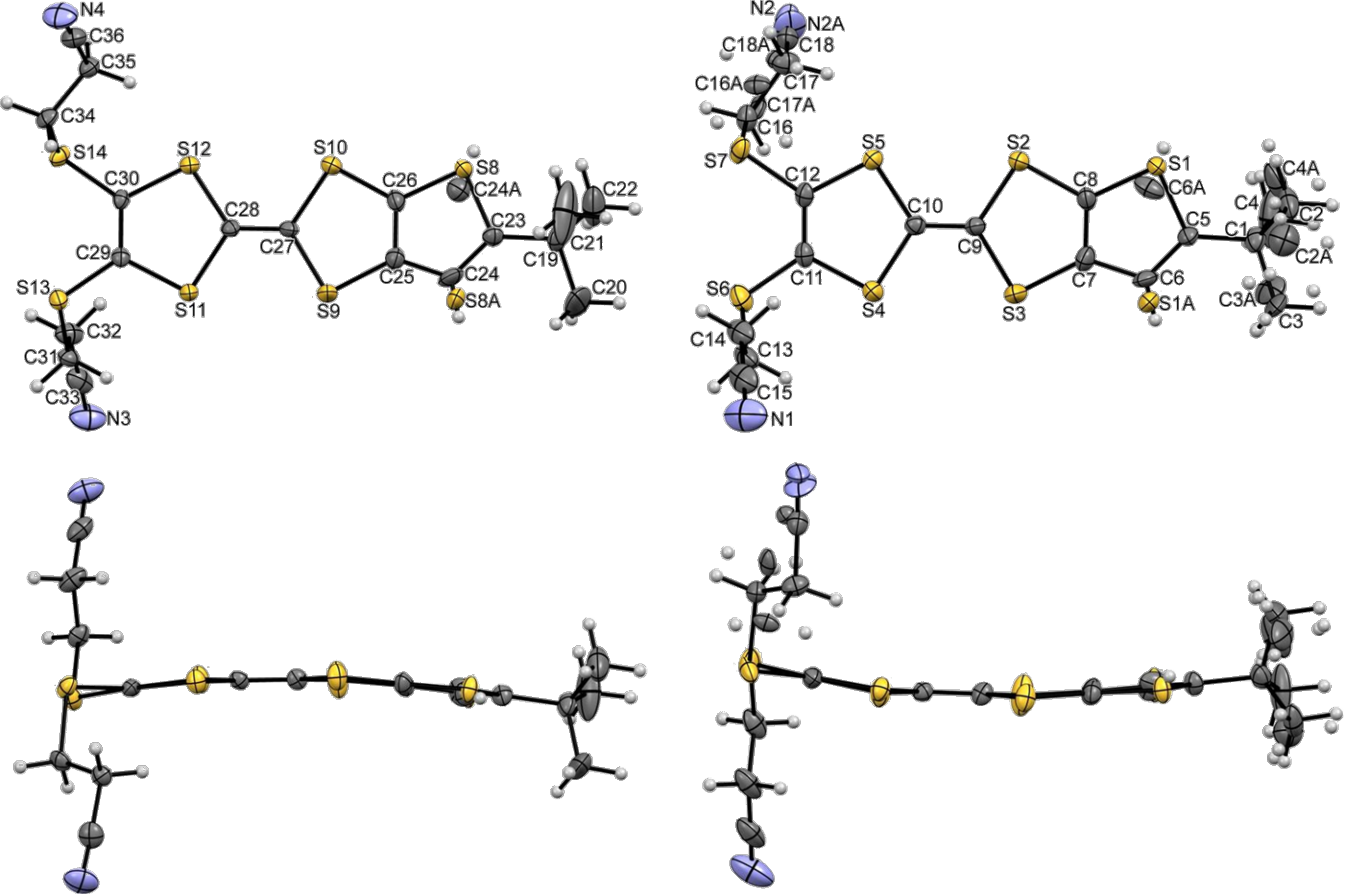
ORTEP views and atomic numbering scheme of α-tbtdt (**1**) with thermal ellipsoids at 50% probability level.

Compound **1** crystallises in the triclinic system, space group *P*−1. The unit cell contains two independent neutral α-tbtdt molecules that present a slightly boat type distortion of the TTF core. The cyanoethyl groups point in opposite directions of the molecules mean plane (Figure 5). The terminal thiophenic sulfur atoms present, in both molecules, an orientation disorder with occupation factors of 77 and 23% for the pair S8/S8A and 71 and 29% for the pair S1/S1A. The bond lengths of the molecule are within the expected range of values for neutral TTF derivatives [11].

**Figure 6:**
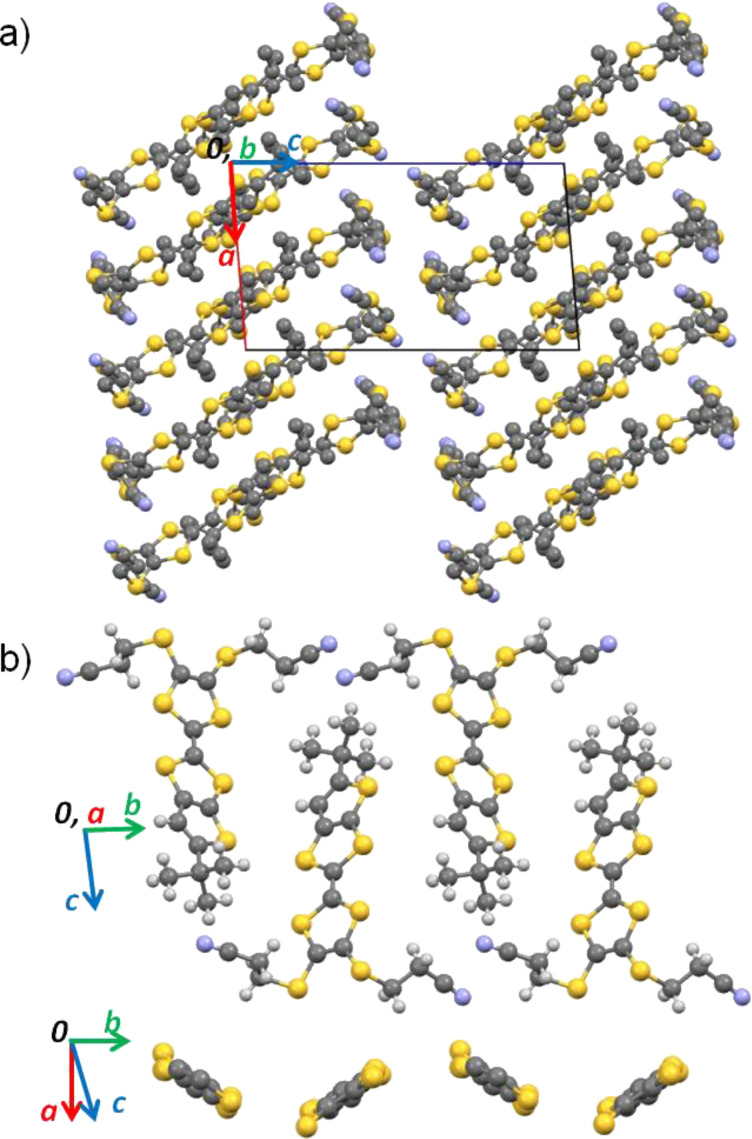
Crystal structure of **1** a) view along the *b* axis*;* b) Partial view of one chain in the *b,c* plane (top) and of the same chain showing the tilt between the molecules along the chain direction (bottom – the cyanoethyl and *tert*-butyl groups were omitted for clarity).

The crystal structure is composed of layers of side-by-side chains of neutral α-tbtdt molecules. Within the chain the molecules are arranged head-to-tail, in a fashion that the cyanoethyl groups point outside the chain and the mean plane of neighbouring molecules is rotated by about 62°. The molecules are connected by several short S^…^S contacts. Besides these contacts the molecules interact upwards and downwards with other molecules in the same layers, and also with the molecules in the neighbouring layers through short S^…^S interactions and N^…^H–C and S^…^H–C hydrogen bonds (Figure 6).

#### (α-mtdt)[Au(mnt)_2_] (**3**)

**Figure 7:**
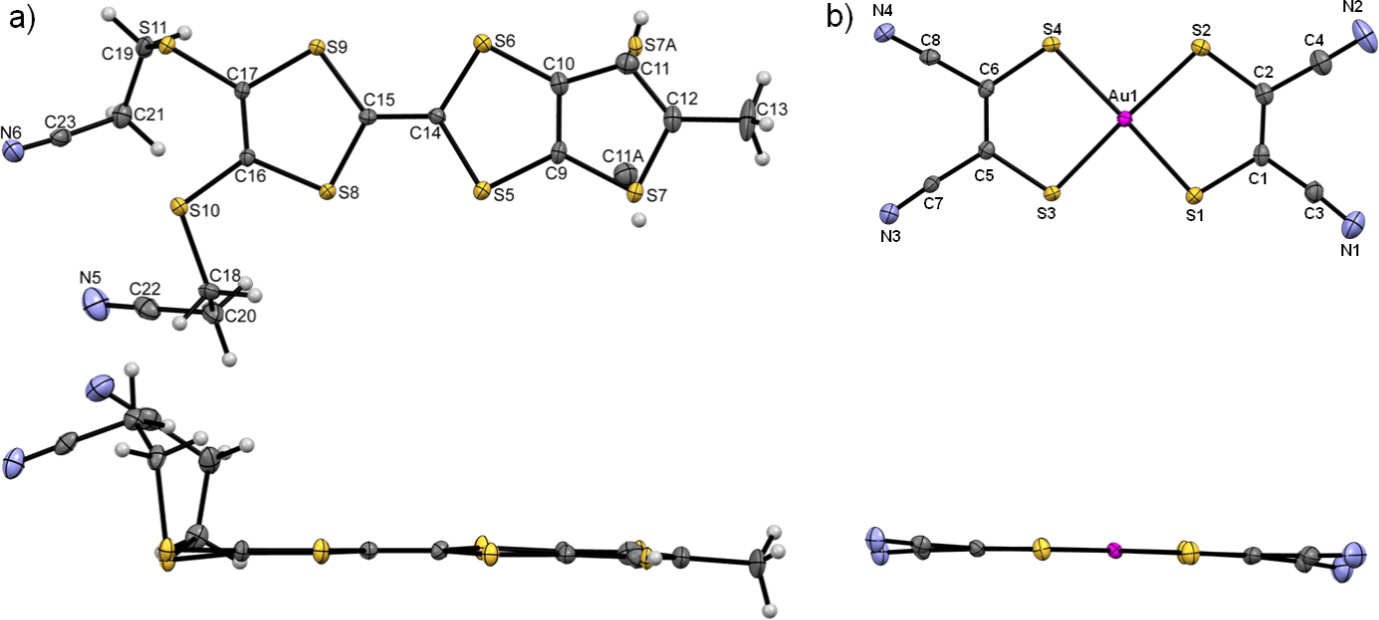
ORTEP views and atomic numbering scheme of a) (α-mtdt)^+^ and b) [Au(mnt)_2_ ]^−^ in compound **3**, with thermal ellipsoids at 50% probability level.

Compound **3** crystallises in the triclinic system, space group *P*−1. The unit cell is composed by one anion [Au(mnt)_2_]^−^ and one fully oxidised donor [α-mtdt]^+^. The donor presents orientation disorder as denoted by the sulfur atom position of the terminal thiophenic ring, with occupation factors of 55% and 45% for S7 and S7A, respectively. The [α-mtdt]^+^ cation is almost planar, with exception of the –(CH_2_)_2_–CN groups, that point out in the same direction (Figure 7). In both donor and acceptor units, the bond lengths are within the expected range, for monoanionic gold dithiolene complexes [21] and fully oxidised TTF donors [18], namely the Au–S and central C14=C15 double bonds (*d*_Au-S_ = 2.392(7) Å, *d*_C14=C15_ = 1.393(4) Å).

**Figure 8:**
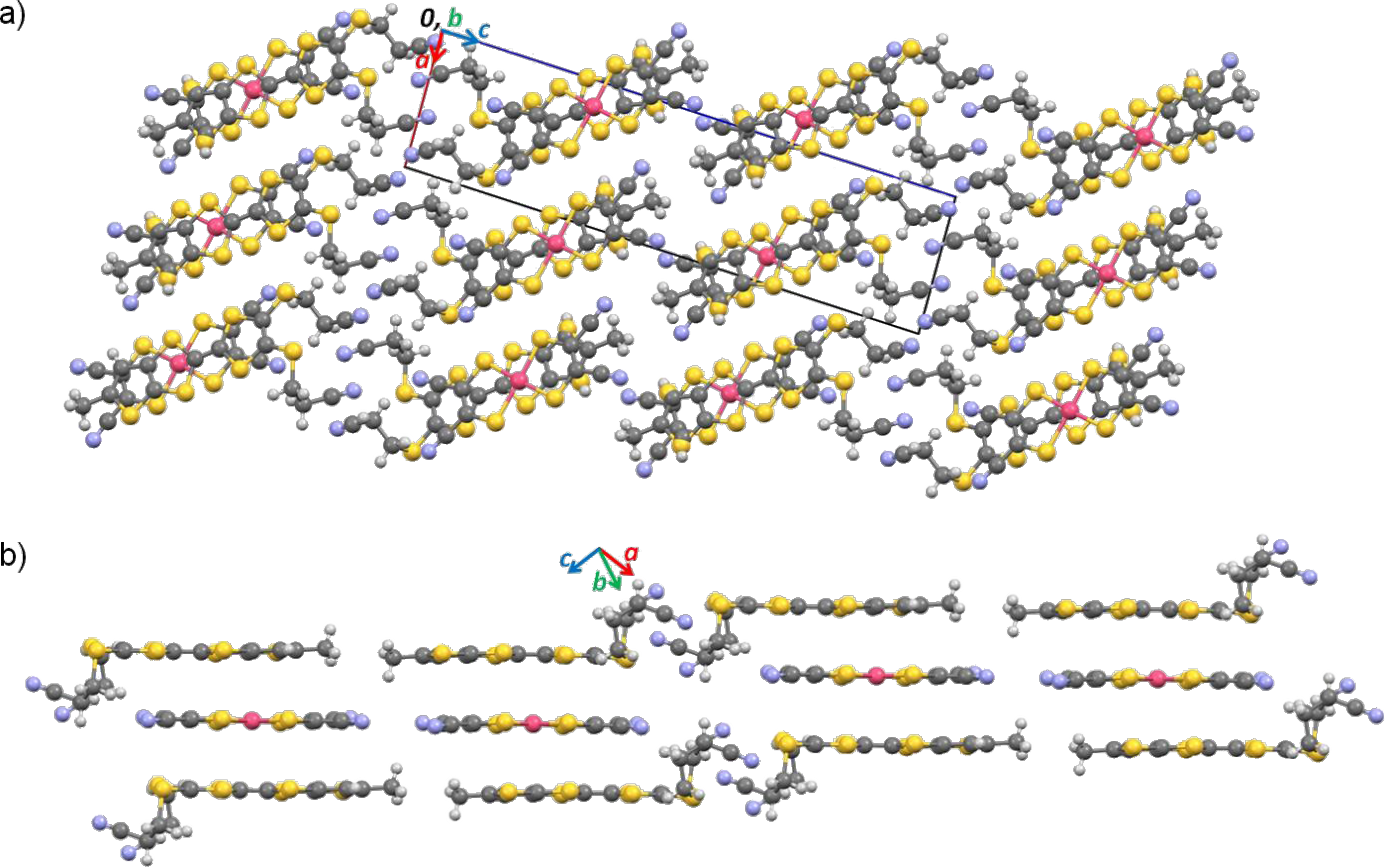
Crystal structure of **3** a) viewed along the *b* axis and b) partial view showing the alternated A–D–A–D stacks and the segregation between the methyl and cyanoethyl groups.

**Figure 9:**
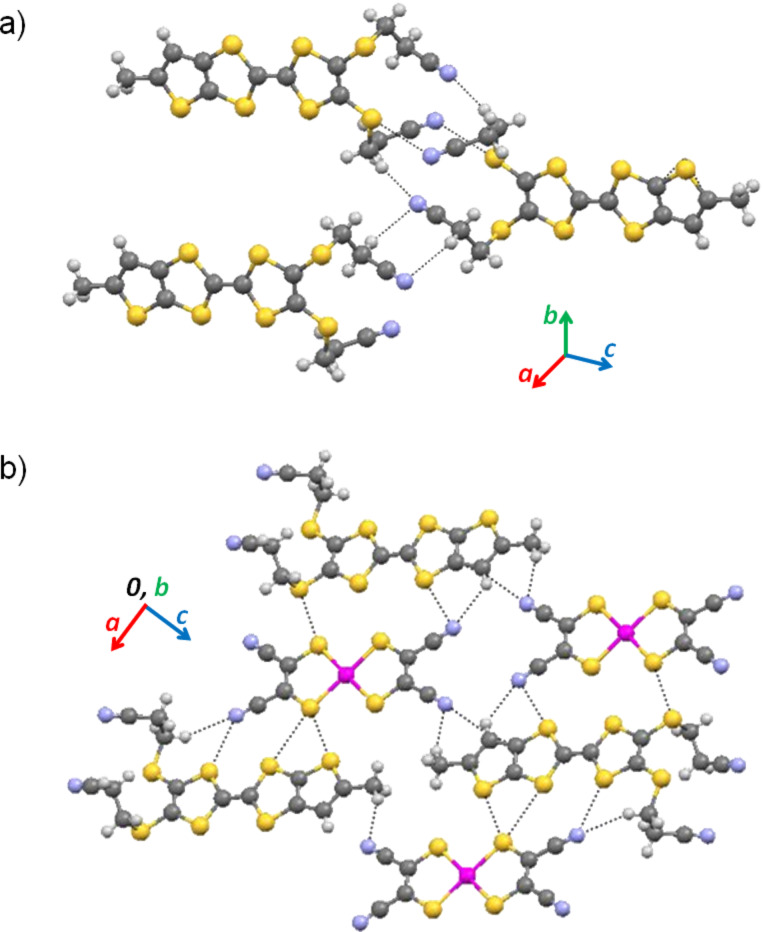
Detail of the crystal structure of **3** showing the short contacts between stacks.

The crystal structure of (α-mtdt)[Au(mnt)_2_] (**3**) is composed of alternated stacks of donor (D)-acceptor (A) molecules, along the *b* axis, with no short contacts between molecules along the stack due to the bulky cyanoethyl and methyl group which prevent a shorter distance between molecular planes. Within the stacks the donor molecules are head-to-head (Figure 8). The interactions between stacks are made in two distinct ways along the molecules long axis: 1) In one side the donor molecules interact, with each other, through the cyanoethyl groups, by C–H^…^N hydrogen bonds and S^…^N short contacts. There are no interactions between A and D molecules and the stacks are out-of-registry; 2) on the other side the stacking interaction is between the nitrile groups of A and the methyl groups of D, and is mediated by a weak C–H^…^N hydrogen bond. In this case the stacks are in-registry (Figure 9).

Along the molecules minor axis the stacks are connected by lateral A–D–A–D S^…^S and N^…^S short interactions.

## Conclusion

In conclusion two new dithiolene ligand precursors, containing fused TTF and alkyl thiophenic moieties were described. These alkyl substituted compounds present a redox behavior with two processes typical of TTF-type donors with lower oxidation potentials compared with unsubstituted analogues. These molecules can also be used as ligand precursors and are expected to open the way to the preparation of extended dithiolene complexes with increased solubility, which may be processed from solution.

## Experimental

### Synthesis

All procedures were performed under inert atmosphere, in nitrogen or argon, unless stated otherwise. All solvents were dried according to the standard literature procedures. Compound 5,6-thieno[2,3-*d*]-1,3-dithiol-2-one (**i**) was obtained following the procedure used by Belo and co-workers [21]. Compounds **II** and **IV** were obtained following the literature procedures [16]. 5-Methylthieno[2,3-*d*][1,3]dithiole-2-thione (**III**) was synthesized as previously described [22]. Compounds 3,3'-{[2-(5-(*tert*-butyl)thieno[2,3-*d*][1,3]dithiol-2-ylidene)-1,3-dithiole-4,5-diyl]bis[sulfanediyl]}dipropanenitrile (**1**) and 3,3'-{[2-(5-methylthieno[2,3-*d*][1,3]dithiol-2-ylidene)-1,3-dithiole-4,5-diyl]bis[sulfanediyl]}dipropanenitrile (**2**), were prepared by an analogous procedure to that in [11]. The tetrabuthylammonium salt of [Au(mnt)_2_]^−^ was synthesized and purified as previously described [23]. All other reagents were reagent grade and used as commercially supplied. Column chromatography was carried out using silica gel (0.063 ± 0.2 mm) from SDS. Elemental analyses of the compounds were performed using an EA 110 CE Instruments automatic analyzer. Melting points were studied on a Stuart Scientific SMP2. IR spectra were obtained on a Bruker FTIR Tensor 27 spectrophotometer. ^1^H NMR and ^13^C NMR spectra were recorded on a Bruker Avance 300 (300 MHz for ^1^H) amd a Bruker Avance 400 (100 MHz for ^13^C) with CDCl_3_ and CD_2_Cl_2_ used as solvents and TMS the internal reference. UV–vis spectra were recorded on a UV-1800 Shimadzu spectrophotometer. Mass spectra were obtained in QIT/MS Bruker HCT by collision-induced dissociation (CID).

**5-(*tert*-Butyl)thieno[2,3-*d*][1,3]dithiol-2-one (I):** 0.536 g of aluminum chloride in 12 mL dichloromethane was cooled to −78 °C in nitrogen atmosphere and a mixture of 0.5 g 5,6-thieno[2,3-*d*][1,3]dithiol-2-one (**i**) and 0.21 mL *t*-BuCl in 6 mL dichloromethane was added dropwise. After stirring for 1 h, the reaction mixture was allowed to warm up to −10 °C and stirred for 3 h, maintaining the temperature between −10 and −5 °C. The product was poured into ice water, neutralized with sodium hydrogen carbonate and extracted with dichloromethane. After drying with magnesium sulfate, the solvent was evaporated. The crude product was purified by column chromatography using *n*-hexane/ethyl acetate (10:1) as eluent and further recrystallized from *n*-hexane to yield 0.616 g (93%) of **I** as white needles. Anal. calcd for C_9_H_10_OS_3_: C, 46.93; H, 4.38; S, 41.75; found: C, 46.74; H, 4.88; S,42.11; ^1^H NMR (300 MHz, CDCl_3_) δ 6.84 (s, 1H), 1.40 (s, 9H); ^13^C NMR (75 MHz, CDCl_3_) δ 194.54 (*C*=O), 159.78 (*C*-C(CH_3_)_3_), 126.06 (Ar), 120.02 (Ar), 115.92 (Ar), 35.44 (*C*(CH_3_)_3_), 32.43 (C(*C*H_3_)_3_).

**3,3'-{[2-(5-(*tert*-Butyl)thieno[2,3-*d*][1,3]dithiol-2-ylidene)-1,3-dithiole-4,5-diyl]bis[sulfanediyl]}dipropanenitrile, α-tbtdt (1):** Compound **I** (300 mg, 1.30 mmol) and compound **II** (395.85 mg, 1.30 mmol) were dissolved in 30 mL of freshly distilled P(OEt)_3_ in a 50 mL round bottomed flask. The mixture was heated to 120 °C for 24 h. Upon cooling 100 mL of methanol was added and the mixture was cooled to 20 °C for 24 h. An orange precipitate was recovered by filtration and washed with cold methanol (3 × 20 mL). The solid was furthermore purified by column chromatography using CH_2_Cl_2_ (*R*_f_ = 0.47). Single crystals were obtained by slow evaporation of a chloroform solution of **1**. Yield: 28% (176 mg); Anal. calcd for C_18_H_18_N_2_S_7_: C, 44.41; H, 3.72; N, 5.75; S, 46.11; found: C, 44.53; H, 4.00; N, 5.17; S, 46.33; FTIR (KBr): 2960 (m, –CH_2_–), 2252 (m, –C≡N), 1634 (m, C=C), 1423 (S, S–CH_2_–R) cm^−1^; ^1^H NMR (300 MHz, CDCl_3_) δ 6.61 (s, 1H, –C=CH–C–), 3.10 (t, *J* = 7.07 Hz, 4H, S–CH_2_–CH_2_–CN), 2.74 (t, *J* = 7.06 Hz, 4H, S–CH_2_–CH_2_–CN), 1.35 (s, 3H, C–(CH_3_)_3_; ^13^C NMR (100 MHz, CD_2_Cl_2_) δ 162.47, 131.18, 128.57, 128.44, 123.76, 121.42, 118.00, 115.58 (C6), 108.47, 35.91, 32.27 (C2, C3, C4), 31.83 (C14, C17), 19.26 (C13, C16); UV (CH_2_Cl_2_) λ_max_, nm: 334, 305.5; MS *m*/*z* (% relative intensity): 485.9 (M^+^, 100); mp 216–217 °C.

**3,3'-{[2-(5-Methylthieno[2,3-*d*][1,3]dithiol-2-ylidene)-1,3-dithiole-4,5-diyl]bis[sulfanediyl]}dipropanenitrile, α-mtdt (2):** Compound **III** (1.000 g, 4.89 mmol) and compound **IV** (1.468 g, 5.09 mmol) were dissolved in 12 mL of P(OMe)_3_ in a 50 mL round bottomed flask and stirred for 24 h at 75/80 °C. After cooling to room temperature, 60 mL of methanol was added to the mixture and further cooled down to 15 °C for another 24 h. The orange precipitate was filtered and washed with 3 × 10 mL of methanol and dried under vacuum. This product was purified by column chromatography using CH_2_Cl_2_ as a solvent (*R*_f_ = 0.62). Recrystallization from *n*-hexane/CH_2_Cl_2_ (3:1) yielded pure **2** (1.38 g, 63%). Anal. calcd for C_15_H_12_N_2_S_7_: C, 40.51; H, 2.72; N, 6.30; S, 50.47; found: C, 40.72; H, 2.89; N, 5.97; S, 51.07; FTIR (KBr) 2924 (m, –CH_2_–), 2251 (m, –C≡N), 1654 and 1421 (m, C=C), 1425 (S, S–CH_2_–R) cm^−1^; ^1^H NMR (300 MHz, CDCl_3_) δ 7.26 (s, 1H, –C=CH–C–), 3.10 (t, *J* = 7.15 Hz, 4H, S–CH_2_–CH_2_–CN), 2.74 (t, *J* = 6.95 Hz, 4H, S–CH_2_–CH_2_–CN), 1.55 (s, 3H, C–CH_3_), ^13^C NMR (100 MHz CD_2_Cl_2_) δ 144.49, 131.43, 128.55, 128.44, 123.82, 121.22, 119.00 (C6), 118.00, 108.43, 31.82 (C20, C21), 19.25 (C18, C19), 16.35 (C13); UV (CH_2_Cl_2_) λ_max,_ nm: 335, 307; MS: *m*/*z* (% relative intensity): 443.9 (M^+^, 100); mp 166–167 °C.

**(α-mtdt)[Au(mnt)_2_] (3):** Crystals were obtained by electrocrystallisation in a manner analogous to the procedure described in [24]. A dichloromethane solution of **2** and *n*-Bu_4_N[Au(mnt)_2_], in approximately stoichiometric amounts, was added to the H-shaped cell, with Pt electrodes and in galvanostatic conditions. Dichloromethane was also purified using standard procedures and freshly distilled immediately before its use. The system was sealed under nitrogen and after ca. 3 days, using a current density of 1 μA·cm^−2^, dark brown plate-shaped crystals were collected in the anode and washed with dichloromethane.

### Redox properties

Cyclic voltammetry data were obtained using a BAS C3 Cell Stand. The voltammograms were obtained at room temperature with a scan rate of 100 mV/s, platinum wire working and counter electrodes and a Ag/AgNO_3_ reference electrode. The measurements were performed on fresh solutions with a concentration of 10^−3^ M, in CH_2_Cl_2_, that contained *n*-Bu_4_NPF_6_ (10^−1^ M) as the supporting electrolyte.

### Crystal structure determination

X-ray diffraction studies were performed with a Bruker APEX-II CCD detector diffractometer using graphite-monochromated Mo Kα radiation (λ = 0.71073 Å), in the φ and ω scans mode. A semi empirical absorption correction was carried out using SADABS [25]. Data collection, cell refinement and data reduction were done with the SMART and SAINT programs [26]. The structures were solved by direct methods using SIR97 [27] and refined by fullmatrix least-squares methods using the program SHELXL97 [28] using the winGX software package [29]. Non-hydrogen atoms were refined with anisotropic thermal parameters whereas H-atoms were placed in idealised positions and allowed to refine riding on the parent C atom. Molecular graphics were prepared using ORTEP 3 [30].

**Crystal data and structure refinement for I:** C_9_H_10_OS_3_, *M* = 230.35 g·mol^−1^, crystal size: 0.30 × 0.20 × 0.10 mm, orthorhombic, space group: *P*2_1_2_1_2_1_, *a* = 9.4587(5) Å, *b* = 9.6256(4) Å, *c* = 11.5993(6) Å, α = β = γ = 90.00°, *V* = 1056.07(9) Å^3^, *Z* = 4, ρ_calc_ = 1.449 g/cm^3^, μ = 0.658 mm^−1^, λ = 0.71073 Å, *T* = 150(2) K, θ range = 2.75–25.26°, reflections collected: 5280, independent: 1897 (*R*_int_ = 0.0294), 140 parameters. The structure was solved by direct methods and refined by full-matrix least squares on F^2^; final *R* indices [I > 2sigma(I)]: *R*_1_ = 0.0329, ω*R*_2_ = 0.0767. CCDC 1051179.

**Crystal data and structure refinement for compound 1 (α-tbtdt):** C_18_H_18_N_2_S_7_, *M* = 486.76 g·mol^−1^, crystal size: 0.50 × 0.20 × 0.02 mm, triclinic, space group: *P*−1, *a* = 9.9526(3) Å, *b* = 12.2499(3) Å, *c* = 17.9352(4) Å, α = 81.3810(10)°, β = 85.234(2)°, γ = 89.2040(10)°, *V* = 2154.46(10) Å^3^, *Z* = 4, ρ_calc_ = 1.501g/cm^3^, μ = 0.739 mm^−1^, λ = 0.71073 Å, *T* = 150(2) K, θ range = 2.93–25.68°, reflections collected: 27683, independent: 8009 (*R*_int_ =0.0482), 599 parameters. The structure was solved by direct methods and refined by full-matrix least squares on F^2^; final *R* indices [I > 2sigma(I)]: *R*_1_ = 0.0387, ω*R*_2_ = 0.0828. CCDC 1051177.

**Crystal data and structure refinement for compound 3 ((α-mtdt)[Au(mnt)_2_]):** C_23_H_12_AuN_6_S_11_, *M* = 922.01 g·mol^−1^, crystal size: 0.40 × 0.10 × 0.04 mm, triclinic, space group: *P*−1, *a* = 7.25300(10) Å, *b* = 8.38770(10) Å, *c* = 26.2705(4) Å, α = 86.2350(10)°, β = 86.1760(10)°, γ = 71.7200(10)°, *V* = 1512.47(4) Å^3^, *Z* = 2, ρ_calc_ = 2.025 g/cm^3^, μ = 5.652 mm^−1^, λ = 0.71073 Å, *T* = 150(2) K, θ range = 2.56**–**26.37°, reflections collected: 25691, independent: 6102 (*R*_int_ = 0.0262), 390 parameters. The structure was solved by direct methods and refined by full-matrix least squares on F^2^; final *R* indices [I > 2sigma(I)]: *R*_1_ = 0.0173, ω*R*_2_ = 0.0420. CCDC 1051178.

## Supporting Information

File 1 Tables of the selected short contacts and hydrogen bonds in the crystal structure of compounds **I**, **1** and **3**.

File 2 CIF file of compound **I**.

File 3 CIF file of compound **1**.

File 4 CIF file of compound **3**.

File 5 CheckCif for compounds **I**, **1** and **3**.
