# Supplementary material for: TTFs nonsymmetrically fused with alkylthiophenic moieties
Source: Beilstein J Org Chem. 2015 May 5;11:628–37. doi: 10.3762/bjoc.11.71 (PMC4464422; doi:10.3762/bjoc.11.71)
Supplement: File 1 — Tables of the selected short contacts and hydrogen bonds in the crystal structure of compounds I, 1 and 3. [file Beilstein_J_Org_Chem-11-628-s001.pdf]

Supporting Information

for

TTFs nonsymmetrically fused with alkylthiophenic moieties

Rafaela A. L. Silva, Bruno J. C. Vieira, Marta M. Andrade, Isabel C. Santos, Sandra Rabaça, Dulce Belo* and Manuel Almeida*

Address: Centro de Ciências e Tecnologias Nucleares, Campus Tecnológico e Nuclear, Instituto Superior Técnico, Universidade de Lisboa, Estrada Nacional 10, ao km 139,7, 2695-066 Bobadela LRS, Portugal

Email: Dulce Belo - dbelo@ctn.ist.utl.pt; Manuel Almeida - malmeida@ctn.ist.utl.pt

* Corresponding author

Tables of the selected short contacts and hydrogen bonds in the crystal structure of compounds I, 1 and 3

Compound I

Table S1: Selected short contacts in the crystal structure of compound **I**.

	Symm. Opp.	Distance (Å)	Interaction
O1···S2	2-x,-1/2+y,1/2-z	3.276(2)	Between bi-chains
O1···S3	2-x,-1/2+y,1/2-z	3.107(4)	Between bi-chains
S2···S3	2-x,-1/2+y,1/2-z	3.412(3)	Between bi-chains

Compound 1

Table S2: Selected short contacts and hydrogen bonds in the crystal structure of α-tbtdt (**1**).

	Symm. Opp.	Distance (Å)	Angle (º)	Interaction
S3···S8	1-x,2-y,-z	3.522(1)		Between chains
S1···S9	1-x,1-y,-z	3.567(2)		Between chains
N1···H17A-C17	x,y,z	2.512(1)	131.14(3)	Between chains
S13···S7	-x,1-y,1-z	3.402(1)		Different Layers
S14···H16B-C16	-x,1-y,1-z	2.9078(6)	161.8(3)	Different Layers
N4···H14B-C14	-x,2-y,1-z	2.607(3)	139.5(2)	Different Layers
S13···H16B-C16	-x,1-y,1-z	2.9165(7)	126.8(3)	Different Layers
S1···S2	1-x,1-y,-z	3.567(2)		Chains in the same layer
S2···S2	1-x,1-y,-z	3.394(1)		Chains in the same layer
S10···S8	-x,2-y,-z	3.529(1)		Chains in the same layer
S10···S10	-x,2-y,-z	3.364(1)		Chains in the same layer
S14···H13B-C13	-1+x,y,z	2.967(2)	132.19(2)	Chains in the same layer
S4···H32B-C32	x,y,z	2.975(3)	126.63(3)	Chains in the same layer
S7···H31A-C31	x,y,z	2.993(3)	124.75(3)	Chains in the same layer
S11···H14A-C14	x,y,z	2.913(1)	129.11(2)	Chains in the same layer
S7···H31A-C31	x,y,z	2.993(2)	124.75(2)	Chains in the same layer
N2···H35B-C35	x,y,z	2.714(1)	132.07(2)	Chains in the same layer
N1···H35A-C35	x,y,z	2,716(3)	121.54(3)	Chains in the same layer
N3···H35-C35	1+x,y,z	2.714(3)	128.31(2)	Chains in the same layer

Compound 3

Table S3: Selected short contacts and hydrogen bonds in the crystal structure of α -mtdt (**3**).

	Distance (Å)	Angle (º)	Symm. Opp.	Interaction
S3···S11	3.5677(9)		-1+x,y,z	A-D[a]
S4···S5	3.5807(8)		1+x,-1+y,z	A-D[a]
S4···S7	3.465(2)		1+x,-1+y,z	A-D[a]
S8···N4	3.195(2)		1+x,-1+y,z	A-D[a]
N1···S6	3.314(3)		-1+x,y,z	A-D[a]
N1···H11-C11	2.689(3)	130.6(6)	-1+x,y,z	A-D[a]
N4···H18A-C18	2.698(2)	148.5(2)	1+x,-1+y,z	A-D[a]
N3···H21B-C21B	2.645(2)	128.45(2)	x,y,z	A-D[b]
N2···H13A-C13	2.6737(3)	152.9(2)	-x,-y,1-z	A-D[b]
N4···H19B-C19	2.712(3)	158.48(2)	x,y,z	A-D[b]
N6···H21A-C21	2.676(2)	118.9(2)	2-x,1-y,-z	D-D[c]
C18···N6	3.230(3)		1-x,2-y,-z	D-D[c]
N5···S10	3.227(3)		1-x,2-y,-z	D-D[c]
N6···H18B-C18	2.421(3)	130.5(2)	1-x,2-y,-z	D-D[c]

[a]Between chains; [b]between chains in the same layers; [c]between chains in neighboring layers.
