# Supplementary material for: TTFs nonsymmetrically fused with alkylthiophenic moieties
Source: Beilstein J Org Chem. 2015 May 5;11:628–37. doi: 10.3762/bjoc.11.71 (PMC4464422; doi:10.3762/bjoc.11.71)

checkCIF/PLATON report

You have not supplied any structure factors. As a result the full set of tests cannot be run.

THIS REPORT IS FOR GUIDANCE ONLY. IF USED AS PART OF A REVIEW PROCEDURE FOR PUBLICATION, IT SHOULD NOT REPLACE THE EXPERTISE OF AN EXPERIENCED CRYSTALLOGRAPHIC REFEREE.

No syntax errors found. CIF dictionary Interpreting this report

Datablock: CCDC-1051179

```
Bond precision:  C-C = 0.0046 A          Wavelength=0.71073

Cell:             a=9.4587(5)      b=9.6256(4)      c=11.5993(6)
                  alpha=90         beta=90          gamma=90
Temperature:      150 K

                Calculated                 Reported
Volume          1056.07(9)                 1056.07(9)
Space group     P 21 21 21                 P2(1)2(1)2(
Hall group      P 2ac 2ab                  ?
Moiety formula  C9 H10 O S3                ?
Sum formula     C9 H10 O S3                C9 H10 O S3
Mr              230.35                     230.35
Dx,g cm-3       1.449                      1.449
Z               4                          4
Mu (mm-1)       0.658                      0.658
F000            480.0                      480.0
F000'           481.62
h,k,lmax        11,11,13                   11,11,13
Nref            1910[ 1122]                1897
Tmin,Tmax       0.854,0.936                0.827,0.937
Tmin'           0.821

Correction method= # Reported T Limits: Tmin=0.827 Tmax=0.937
AbsCorr = MULTI-SCAN

Data completeness= 1.69/0.99      Theta(max)= 25.260

R(reflections)= 0.0329( 1690)     wR2(reflections)= 0.0793( 1897)

S = 1.075                   Npar= 140
```

The following ALERTS were generated. Each ALERT has the format
test-name_ALERT_alert-type_alert-level.
Click on the hyperlinks for more details of the test.

Alert level B

```
PLAT230_ALERT_2_B Hirshfeld Test Diff for    C6     --  C8      ..        7.3 su
```

Alert level C

```
PLAT213_ALERT_2_C Atom C4A             has ADP max/min Ratio .....        3.3 oblate
PLAT234_ALERT_4_C Large Hirshfeld Difference C5     --  C4      ..       0.17 Ang.
PLAT242_ALERT_2_C Low       Ueq as Compared to Neighbors for .....         C6 Check
PLAT340_ALERT_3_C Low Bond Precision on  C-C Bonds ...............     0.0046 Ang.
```

Alert level G

```
PLAT005_ALERT_5_G No _iucr_refine_instructions_details  in the CIF     Please Do !
PLAT066_ALERT_1_G Predicted and Reported Tmin&Tmax Range Identical          ? Check
PLAT230_ALERT_2_G Hirshfeld Test Diff for    C4A    --  C5      ..        6.3 su
PLAT300_ALERT_4_G Atom Site Occupancy of >S3A    is Constrained at      0.511 Check
PLAT300_ALERT_4_G Atom Site Occupancy of <S3     is Constrained at      0.489 Check
PLAT300_ALERT_4_G Atom Site Occupancy of >C4A    is Constrained at      0.511 Check
PLAT300_ALERT_4_G Atom Site Occupancy of <C4     is Constrained at      0.489 Check
PLAT301_ALERT_3_G Main Residue  Disorder ............ Percentage =         15 Note
PLAT779_ALERT_4_G Suspect or Irrelevant (Bond) Angle in CIF .... #         16 Check
              C4A  -C2   -S3      1.555   1.555   1.555               14.80 Deg.
PLAT779_ALERT_4_G Suspect or Irrelevant (Bond) Angle in CIF .... #         24 Check
              C4   -C3   -S3A     1.555   1.555   1.555               14.30 Deg.
PLAT779_ALERT_4_G Suspect or Irrelevant (Bond) Angle in CIF .... #         30 Check
              C4   -C5   -S3A     1.555   1.555   1.555               17.20 Deg.
PLAT779_ALERT_4_G Suspect or Irrelevant (Bond) Angle in CIF .... #         32 Check
              C4A  -C5   -S3      1.555   1.555   1.555               13.80 Deg.
PLAT850_ALERT_4_G Check Flack Parameter Exact Value 0.00 and su ..       0.12 Check
PLAT899_ALERT_4_G SHELXL97   is Deprecated and Succeeded by SHELXL       2014 Note
```

```
   0 ALERT level A = Most likely a serious problem - resolve or explain
   1 ALERT level B = A potentially serious problem, consider carefully
   4 ALERT level C = Check. Ensure it is not caused by an omission or oversight
  14 ALERT level G = General information/check it is not something unexpected

   1 ALERT type 1 CIF construction/syntax error, inconsistent or missing data
   4 ALERT type 2 Indicator that the structure model may be wrong or deficient
   2 ALERT type 3 Indicator that the structure quality may be low
  11 ALERT type 4 Improvement, methodology, query or suggestion
   1 ALERT type 5 Informative message, check
```

Datablock: CCDC-1051177

```
Bond precision:  C-C = 0.0041 A          Wavelength=0.71073

Cell:            a=9.9526(3)       b=12.2499(3)     c=17.9352(4)
                 alpha=81.381(1)   beta=85.234(2)   gamma=89.204(1)
Temperature:     150 K
```

	Calculated	Reported
Volume	2154.46(10)	2154.46(10)
Space group	P -1	P-1
Hall group	-P 1	?
Moiety formula	C18 H18 N2 S7	?
Sum formula	C18 H18 N2 S7	C18 H18 N2 S7
Mr	486.76	486.76
Dx,g cm-3	1.501	1.501
Z	4	4
Mu (mm-1)	0.739	0.739
F000	1008.0	1008.0
F000’	1011.67	
h,k,lmax	12,14,21	12,14,21
Nref	8173	8009
Tmin,Tmax	0.837,0.985	0.709,0.985
Tmin’	0.691	

Correction method= # Reported T Limits: Tmin=0.709 Tmax=0.985
AbsCorr = MULTI-SCAN

Data completeness= 0.980 Theta(max)= 25.680

R(reflections)= 0.0387(5668) wR2(reflections)= 0.0904(8009)

S = 1.019 Npar= 599

The following ALERTS were generated. Each ALERT has the format
test-name_ALERT_alert-type_alert-level.
Click on the hyperlinks for more details of the test.

Alert level C

```
PLAT213_ALERT_2_C Atom C3               has ADP max/min Ratio .....        3.3 prolat
PLAT213_ALERT_2_C Atom C21              has ADP max/min Ratio .....        3.4 prolat
PLAT220_ALERT_2_C Large Non-Solvent  C     Ueq(max)/Ueq(min) Range        4.3 Ratio
PLAT220_ALERT_2_C Large Non-Solvent  C     Ueq(max)/Ueq(min) Range        5.8 Ratio
PLAT222_ALERT_3_C Large Non-Solvent  H     Uiso(max)/Uiso(min) ...        5.3 Ratio
PLAT234_ALERT_4_C Large Hirshfeld Difference C5     --  C6A     ..       0.17 Ang.
PLAT241_ALERT_2_C High      Ueq as Compared to Neighbors for .....         S3 Check
PLAT241_ALERT_2_C High      Ueq as Compared to Neighbors for .....         S9 Check
PLAT242_ALERT_2_C Low       Ueq as Compared to Neighbors for .....         C1 Check
PLAT242_ALERT_2_C Low       Ueq as Compared to Neighbors for .....        C19 Check
PLAT340_ALERT_3_C Low Bond Precision on  C-C Bonds ...............     0.0041 Ang.
```

Alert level G

```
PLAT003_ALERT_2_G Number of Uiso or Uij Restrained non-H Atoms ...          3 Report
PLAT005_ALERT_5_G No _iucr_refine_instructions_details  in the CIF     Please Do !
PLAT230_ALERT_2_G Hirshfeld Test Diff for    C32    --  C33     ..        6.0 su
PLAT230_ALERT_2_G Hirshfeld Test Diff for    C35    --  C36     ..        6.0 su
PLAT300_ALERT_4_G Atom Site Occupancy of >S1     is Constrained at      0.709 Check
PLAT300_ALERT_4_G Atom Site Occupancy of <S1A    is Constrained at      0.291 Check
PLAT300_ALERT_4_G Atom Site Occupancy of >N2     is Constrained at      0.794 Check
```

```
PLAT300_ALERT_4_G Atom Site Occupancy of >C2     is Constrained at      0.725 Check
PLAT300_ALERT_4_G Atom Site Occupancy of >C3     is Constrained at      0.725 Check
PLAT300_ALERT_4_G Atom Site Occupancy of >C4     is Constrained at      0.725 Check
PLAT300_ALERT_4_G Atom Site Occupancy of >C6     is Constrained at      0.709 Check
PLAT300_ALERT_4_G Atom Site Occupancy of >C16    is Constrained at      0.794 Check
PLAT300_ALERT_4_G Atom Site Occupancy of >C17    is Constrained at      0.794 Check
PLAT300_ALERT_4_G Atom Site Occupancy of >C18    is Constrained at      0.794 Check
PLAT300_ALERT_4_G Atom Site Occupancy of <N2A    is Constrained at      0.206 Check
PLAT300_ALERT_4_G Atom Site Occupancy of <C2A    is Constrained at      0.275 Check
PLAT300_ALERT_4_G Atom Site Occupancy of <C3A    is Constrained at      0.275 Check
PLAT300_ALERT_4_G Atom Site Occupancy of <C4A    is Constrained at      0.275 Check
PLAT300_ALERT_4_G Atom Site Occupancy of <C6A    is Constrained at      0.291 Check
PLAT300_ALERT_4_G Atom Site Occupancy of <C16A   is Constrained at      0.206 Check
PLAT300_ALERT_4_G Atom Site Occupancy of <C17A   is Constrained at      0.206 Check
PLAT300_ALERT_4_G Atom Site Occupancy of <C18A   is Constrained at      0.206 Check
PLAT300_ALERT_4_G Atom Site Occupancy of >S8     is Constrained at      0.773 Check
PLAT300_ALERT_4_G Atom Site Occupancy of <S8A    is Constrained at      0.227 Check
PLAT300_ALERT_4_G Atom Site Occupancy of >C24    is Constrained at      0.773 Check
PLAT300_ALERT_4_G Atom Site Occupancy of <C24A   is Constrained at      0.227 Check
PLAT301_ALERT_3_G Main Residue  Disorder ........... Percentage =          20 Note
PLAT720_ALERT_4_G Number of Unusual/Non-Standard Labels ..........          9 Note
PLAT779_ALERT_4_G Suspect or Irrelevant (Bond) Angle in CIF .... #          8 Check
              C16A -S7   -C16     1.555   1.555   1.555             34.60 Deg.
PLAT779_ALERT_4_G Suspect or Irrelevant (Bond) Angle in CIF .... #         18 Check
              C2A  -C1   -C2      1.555   1.555   1.555             33.40 Deg.
PLAT779_ALERT_4_G Suspect or Irrelevant (Bond) Angle in CIF .... #         31 Check
              C4   -C1   -C4A     1.555   1.555   1.555             37.40 Deg.
PLAT779_ALERT_4_G Suspect or Irrelevant (Bond) Angle in CIF .... #         39 Check
              C6   -C5   -S1A     1.555   1.555   1.555             14.20 Deg.
PLAT779_ALERT_4_G Suspect or Irrelevant (Bond) Angle in CIF .... #         43 Check
              C6A  -C5   -S1      1.555   1.555   1.555             15.60 Deg.
PLAT779_ALERT_4_G Suspect or Irrelevant (Bond) Angle in CIF .... #         48 Check
              C6   -C7   -S1A     1.555   1.555   1.555             15.80 Deg.
PLAT779_ALERT_4_G Suspect or Irrelevant (Bond) Angle in CIF .... #         54 Check
              C6A  -C8   -S1      1.555   1.555   1.555             16.80 Deg.
PLAT779_ALERT_4_G Suspect or Irrelevant (Bond) Angle in CIF .... #        110 Check
              C24  -C23  -S8A     1.555   1.555   1.555             15.60 Deg.
PLAT779_ALERT_4_G Suspect or Irrelevant (Bond) Angle in CIF .... #        114 Check
              C24A -C23  -S8      1.555   1.555   1.555             12.90 Deg.
PLAT779_ALERT_4_G Suspect or Irrelevant (Bond) Angle in CIF .... #        119 Check
              C24  -C25  -S8A     1.555   1.555   1.555             14.40 Deg.
PLAT779_ALERT_4_G Suspect or Irrelevant (Bond) Angle in CIF .... #        125 Check
              C24A -C26  -S8      1.555   1.555   1.555             16.70 Deg.
PLAT860_ALERT_3_G Number of Least-Squares Restraints .............         24 Note
PLAT899_ALERT_4_G SHELXL97   is Deprecated and Succeeded by SHELXL       2014 Note
```

---

0 **ALERT level A** = Most likely a serious problem - resolve or explain
0 **ALERT level B** = A potentially serious problem, consider carefully
11 **ALERT level C** = Check. Ensure it is not caused by an omission or oversight
41 **ALERT level G** = General information/check it is not something unexpected

0 ALERT type 1 CIF construction/syntax error, inconsistent or missing data
11 ALERT type 2 Indicator that the structure model may be wrong or deficient
4 ALERT type 3 Indicator that the structure quality may be low
36 ALERT type 4 Improvement, methodology, query or suggestion
1 ALERT type 5 Informative message, check

Datablock: CCDC-1051178

Bond precision: C-C = 0.0041 A Wavelength=0.71073

Cell:	a=7.2530(1)	b=8.3877(1)	c=26.2705(4)
	alpha=86.235(1)	beta=86.176(1)	gamma=71.720(1)
Temperature:	150 K		

	Calculated	Reported
Volume	1512.47(4)	1512.47(4)
Space group	P -1	P-1
Hall group	-P 1	?
Moiety formula	C15 H12 N2 S7, C8 Au N4 S4	C15 H12 N2 S7, C8 Au N4 S4
Sum formula	C23 H12 Au N6 S11	C23 H12 Au N6 S11
Mr	922.02	922.01
Dx,g cm-3	2.025	2.025
Z	2	2
Mu (mm-1)	5.652	5.652
F000	894.0	894.0
F000’	892.98	
h,k,lmax	9,10,32	9,10,32
Nref	6181	6102
Tmin,Tmax	0.513,0.798	0.211,0.805
Tmin’	0.103	

Correction method= # Reported T Limits: Tmin=0.211 Tmax=0.805
AbsCorr = MULTI-SCAN

Data completeness= 0.987 Theta(max)= 26.370

R(reflections)= 0.0173(6057) wR2(reflections)= 0.0421(6102)

S = 1.253 Npar= 390

The following ALERTS were generated. Each ALERT has the format
test-name_ALERT_alert-type_alert-level.
Click on the hyperlinks for more details of the test.

Alert level C

PLAT220_ALERT_2_C Large Non-Solvent C Ueq(max)/Ueq(min) Range 3.2 Ratio

---

Alert level G

PLAT003_ALERT_2_G Number of Uiso or Uij Restrained non-H Atoms ... 2 Report
PLAT005_ALERT_5_G No _iucr_refine_instructions_details in the CIF Please Do !
PLAT154_ALERT_1_G The su’s on the Cell Angles are Equal .... 0.00100 Degree
PLAT230_ALERT_2_G Hirshfeld Test Diff for S7 -- C12 .. 6.1 su
PLAT230_ALERT_2_G Hirshfeld Test Diff for S7A -- C12 .. 5.6 su
PLAT300_ALERT_4_G Atom Site Occupancy of >S7 is Constrained at 0.551 Check
PLAT300_ALERT_4_G Atom Site Occupancy of <S7A is Constrained at 0.449 Check
PLAT300_ALERT_4_G Atom Site Occupancy of >C11 is Constrained at 0.551 Check
PLAT300_ALERT_4_G Atom Site Occupancy of <C11A is Constrained at 0.449 Check

```
PLAT301_ALERT_3_G Main Residue  Disorder ........... Percentage =         5 Note
PLAT779_ALERT_4_G Suspect or Irrelevant (Bond) Angle in CIF .... #        35 Check
              C11A -C9   -S7      1.555   1.555   1.555              9.60 Deg.
PLAT779_ALERT_4_G Suspect or Irrelevant (Bond) Angle in CIF .... #        41 Check
              C11  -C10  -S7A     1.555   1.555   1.555              9.70 Deg.
PLAT779_ALERT_4_G Suspect or Irrelevant (Bond) Angle in CIF .... #        48 Check
              C11  -C12  -S7A     1.555   1.555   1.555             12.10 Deg.
PLAT779_ALERT_4_G Suspect or Irrelevant (Bond) Angle in CIF .... #        52 Check
              C11A -C12  -S7      1.555   1.555   1.555             13.10 Deg.
PLAT860_ALERT_3_G Number of Least-Squares Restraints .............        12 Note
PLAT899_ALERT_4_G SHELXL97   is Deprecated and Succeeded by SHELXL      2014 Note
```

```
   0 ALERT level A = Most likely a serious problem - resolve or explain
   0 ALERT level B = A potentially serious problem, consider carefully
   1 ALERT level C = Check. Ensure it is not caused by an omission or oversight
  16 ALERT level G = General information/check it is not something unexpected

   1 ALERT type 1 CIF construction/syntax error, inconsistent or missing data
   4 ALERT type 2 Indicator that the structure model may be wrong or deficient
   2 ALERT type 3 Indicator that the structure quality may be low
   9 ALERT type 4 Improvement, methodology, query or suggestion
   1 ALERT type 5 Informative message, check
```

It is advisable to attempt to resolve as many as possible of the alerts in all categories. Often the minor alerts point to easily fixed oversights, errors and omissions in your CIF or refinement strategy, so attention to these fine details can be worthwhile. In order to resolve some of the more serious problems it may be necessary to carry out additional measurements or structure refinements. However, the purpose of your study may justify the reported deviations and the more serious of these should normally be commented upon in the discussion or experimental section of a paper or in the "special_details" fields of the CIF. checkCIF was carefully designed to identify outliers and unusual parameters, but every test has its limitations and alerts that are not important in a particular case may appear. Conversely, the absence of alerts does not guarantee there are no aspects of the results needing attention. It is up to the individual to critically assess their own results and, if necessary, seek expert advice.

Publication of your CIF in IUCr journals

A basic structural check has been run on your CIF. These basic checks will be run on all CIFs submitted for publication in IUCr journals (*Acta Crystallographica*, *Journal of Applied Crystallography*, *Journal of Synchrotron Radiation*); however, if you intend to submit to *Acta Crystallographica Section C* or *E*, you should make sure that full publication checks are run on the final version of your CIF prior to submission.

Publication of your CIF in other journals

Please refer to the *Notes for Authors* of the relevant journal for any special instructions relating to CIF submission.

PLATON version of 29/01/2015; check.def file version of 29/01/2015

Datablock CCDC-1051179 - ellipsoid plot

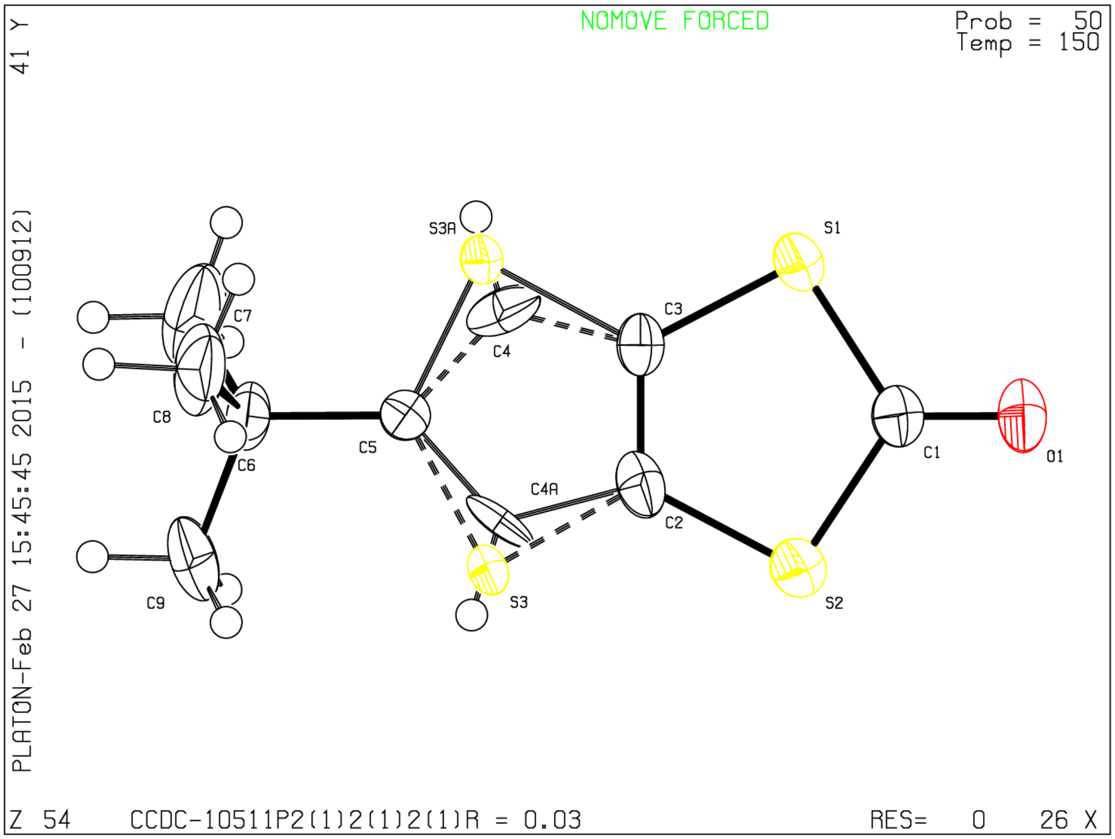

Datablock CCDC-1051177 - ellipsoid plot

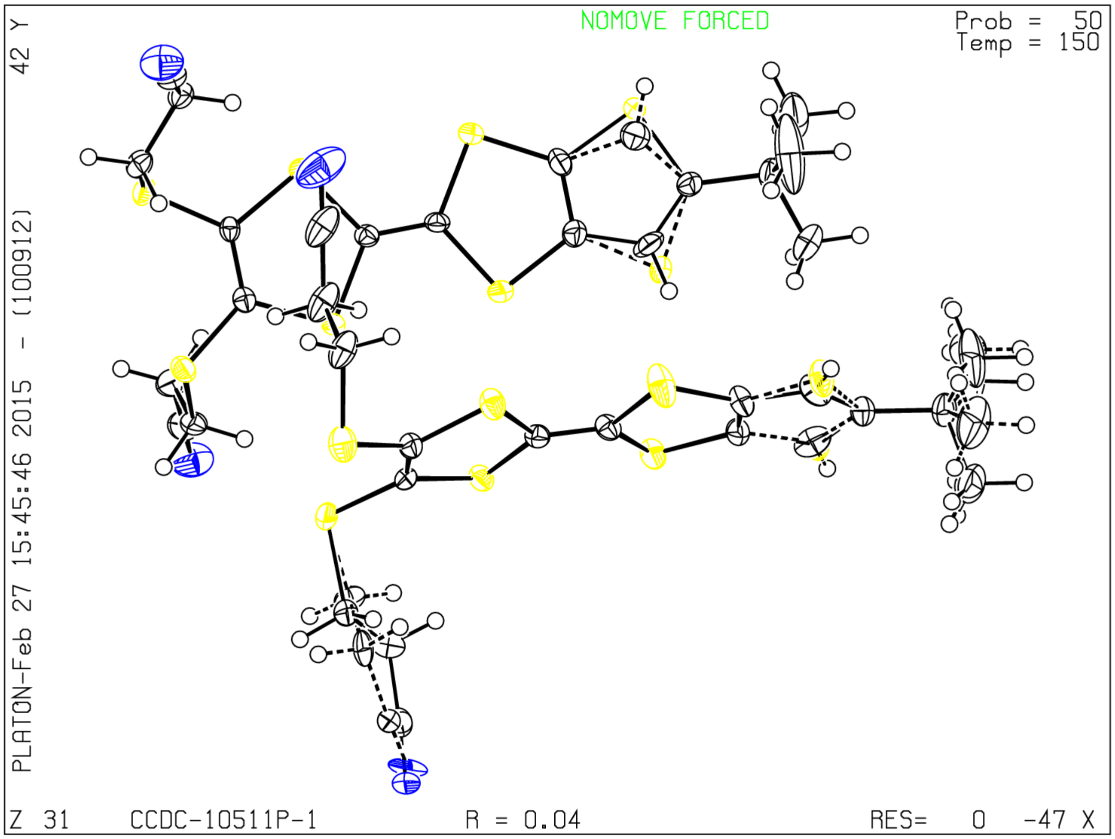

Datablock CCDC-1051178 - ellipsoid plot

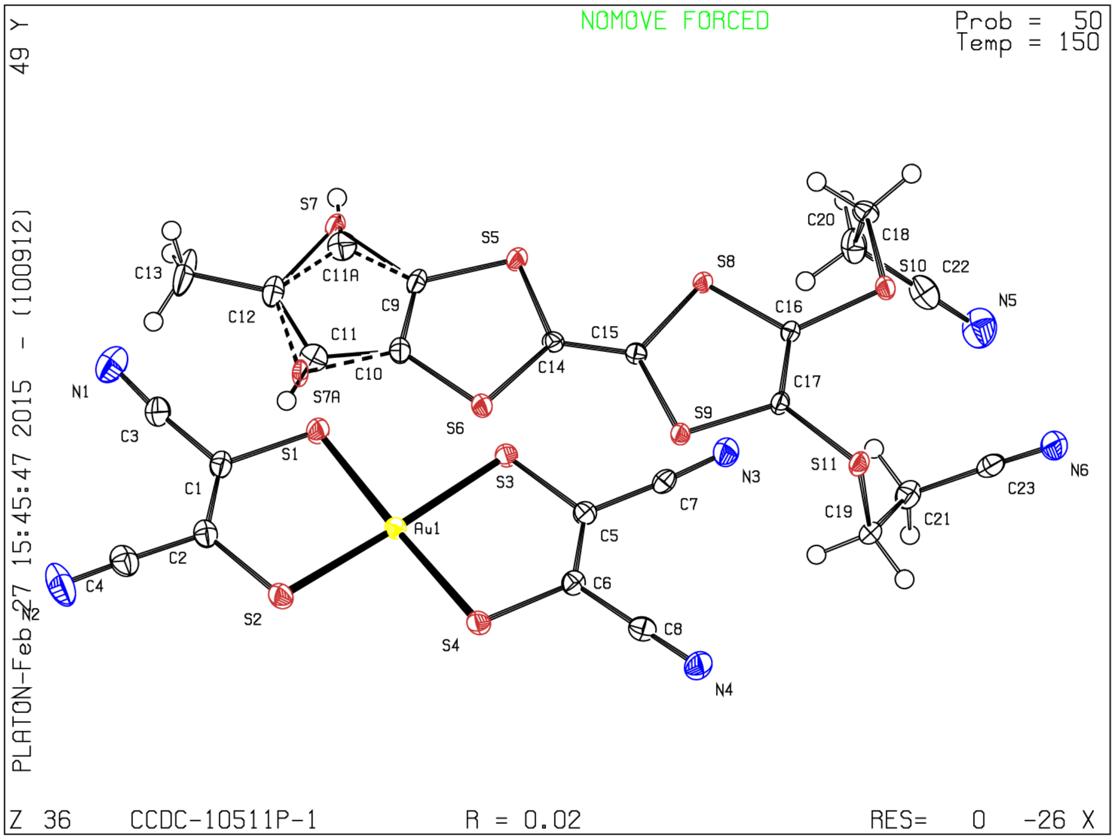

Supplement: File 5 — CheckCif for compounds I, 1 and 3. [file Beilstein_J_Org_Chem-11-628-s005.pdf]
